# Siphophage 0105phi7-2 of *Bacillus thuringiensis*: Novel Propagation, DNA, and Genome-Implied Assembly

**DOI:** 10.3390/ijms24108941

**Published:** 2023-05-18

**Authors:** Samantha M. Roberts, Miranda Aldis, Elena T. Wright, Cara B. Gonzales, Zhao Lai, Susan T. Weintraub, Stephen C. Hardies, Philip Serwer

**Affiliations:** 1 Department of Microbiology, Immunology and Molecular Genetics, UT Health, San Antonio, TX 78229, USA; robertss1@livemail.uthscsa.edu (S.M.R.); aldism@livemail.uthscsa.edu (M.A.); 2 Department of Biochemistry and Structural Biology, UT Health, San Antonio, TX 78229, USA; wrighte@uthscsa.edu (E.T.W.); weintraub@uthscsa.edu (S.T.W.); hardies@uthscsa.edu (S.C.H.); 3 Department of Comprehensive Dentistry, UT Health, San Antonio, TX 78229, USA; gonzalesc5@uthscsa.edu; 4 Department of Molecular Medicine, UT Health, San Antonio, TX 78229, USA; laiz@uthscsa.edu

**Keywords:** ATP, signaling, bacteriophage genomics, bacteriophage, novel, DNA compaction, partial, mass spectrometry, native gel electrophoresis, phage therapy, scaffolding of protein assembly

## Abstract

Diversity of phage propagation, physical properties, and assembly promotes the use of phages in ecological studies and biomedicine. However, observed phage diversity is incomplete. *Bacillus thuringiensis* siphophage, 0105phi-7-2, first described here, significantly expands known phage diversity, as seen via in-plaque propagation, electron microscopy, whole genome sequencing/annotation, protein mass spectrometry, and native gel electrophoresis (AGE). Average plaque diameter vs. plaque-supporting agarose gel concentration plots reveal unusually steep conversion to large plaques as agarose concentration decreases below 0.2%. These large plaques sometimes have small satellites and are made larger by orthovanadate, an ATPase inhibitor. Phage head–host-cell binding is observed by electron microscopy. We hypothesize that this binding causes plaque size-increase via biofilm evolved, ATP stimulated ride-hitching on motile host cells by temporarily inactive phages. Phage 0105phi7-2 does not propagate in liquid culture. Genomic sequencing/annotation reveals history as temperate phage and distant similarity, in a virion-assembly gene cluster, to prototypical siphophage SPP1 of *Bacillus subtilis*. Phage 0105phi7-2 is distinct in (1) absence of head-assembly scaffolding via either separate protein or classically sized, head protein-embedded peptide, (2) producing partially condensed, head-expelled DNA, and (3) having a surface relatively poor in AGE-detected net negative charges, which is possibly correlated with observed low murine blood persistence.

## 1. Introduction

The study of bacteriophages (phages) provides a link between physics and biology [1,2,3]. Biology is differentiated from physics by its inclusion of evolution. Within the context of thermodynamics, evolution generates events that can be interpreted as purposeful and order-generating [4]. Variations in the environment cause variations in microbial evolution, as seen in the evolution of multidrug resistant bacteria for which phages are sought as therapeutics (phage therapy [5,6,7,8,9,10,11,12,13]). Thus, the details of phage propagation are expected to vary in ways that are potentially useful for both understanding evolution and using phages for phage therapy and other areas of biomedicine.

The possibility exists that our current understanding of evolved phage characteristics is limited by the way in which phages have been isolated, most via liquid culture [10,11,12,13]. For example, no membrane-containing, single-stranded RNA phage has been isolated [14]. If these latter phages exist, finding a way to isolate them will provide (1) a more accessible model for understanding common aspects of assembly and (2) a display vaccine-vector with increased physical resemblance to the targeted viral pathogen in the case of epidemic-causing viruses, such as influenza viruses [15] and coronaviruses [16], that are membrane covered and have single-stranded RNA genomes.

Thus, we screened previously isolated, uncharacterized phages for unusual properties. The phages had been isolated and 3× cloned by in-gel propagation only [17,18] and had not been further investigated. Investigation of these phages had stopped, in part, because of limitations of genome sequencing technology, including its cost. These limitations have subsequently been removed (reviews [19,20]).

In the current study, we describe characterization of one of these phages, 0105phi7-2, which is found to be novel in several ways. Novelty begins with the characteristics of in-gel propagation, which suggest that 0105phi7-2 sometimes attaches, in a noninfective state, to its motile host and rides with the host to places of higher intact host concentration. Novelty continues with capsid assembly, expelled DNA conformation, and surface electrical charge on the capsid, the latter a possible indicator of phage persistence in blood. The data suggest previous evolution in biofilms. The details are presented here.

## 2. Results

### 2.1. Initial Screening and Characterization of Newly Isolated Phages

**Figure 1:**
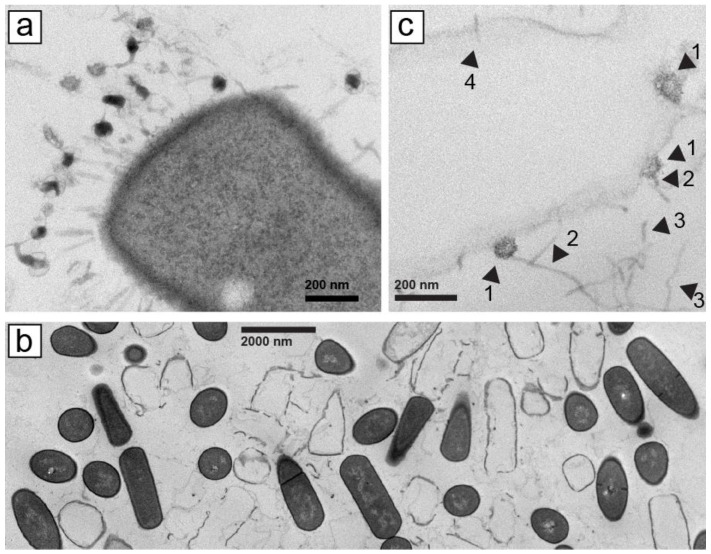
Electron microscopy of thin sections of plaques formed by phages (**a**) 0305phi7-4 and (**b**,**c**) 0105phi7-2. Procedures are described in Section 4. In (**c**), arrowheads are identified in the text.

To efficiently (in time and cost) screen phages in large number (~400 initially unscreened), we began by electron microscopic (EM) screening of thin sections of phage plaques. This procedure has previously been used in non-screening contexts [21,22], but is not in general use for screening. It was effective in identifying myophages (with contractile tails), such as *Bacillus thuringiensis* phage 0305phi7-4 (Figure 1a).

However, with phage 0105phi7-2 (isolated in January of 2005), we initially did not find phage tail-classifying images of phages with tail attached to a host cell, for example, the image in Figure 1a. We chose specimen regions with cells that had been phage infected, as judged by absence of cytoplasm. These “ghost” cells typically still had a cell wall with the shape of a cell. Ghost cells were interspersed with cells that had cytoplasm, the latter with no indication of phage infection. Neither ghost nor intact cells usually had any phages or phage components attached (Figure 1b, magnified, as verified with other more magnified images not shown).

Searching of the specimen revealed some phage head-like objects that were in direct contact with lysed (not un-lysed), ghost host cells; these objects were the size of phage heads seen in Section 2.5 and sometimes had polygonal character (head–ghost-cell contact; Figure 1c, arrowheads 1). No evidence was present of a tail between the head and the host cell (Figure 1c, arrowheads 1). These heads did appear to have a straight, tail-like object that projected away from the cell to which the head was attached (Figure 1c, arrowheads 2). Agarose fibers were also observed. Curvature of agarose fibers (Figure 1c, arrowheads 3) usually distinguished agarose fibers from tails, which were straight. The number of similarly structured heads seen was 20, some not cell attached. No head-like object appeared cell-attached via a tail. The infection had presumably been caused by tail–host-attached phages that left an empty head that was no longer both intact and visible. An apparent infection-initiating tail, without head, is indicated by arrowhead 4 in Figure 1c.

The head–host-cell attachment was initially mysterious. However, this observation made sense when we subsequently characterized 0105phi7-2 and its propagation in Section 2.2, below. We have not performed the analysis of Figure 1 with other siphophages.

### 2.2. Rapid Rise in Plaque Diameter with Decreasing Plaque-Supporting Agarose Gel Percentage, A

**Figure 2:**
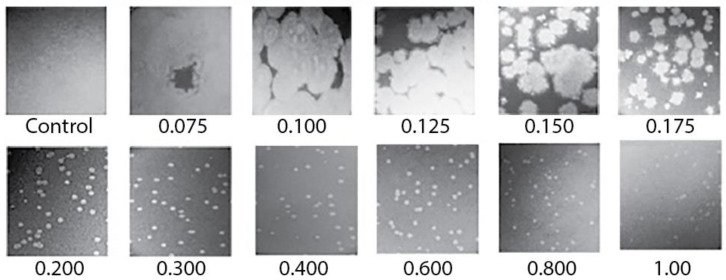
Images of plaques of phage 0105phi7-2 vs. *A*. Petri plates were incubated for 16.0 h at 25 ± 3 °C. The value of *A* (percentage of agarose) is indicated beneath the panel. Control indicates a plate with the host, but no phage inoculated.

We had difficulty discerning plaques when we initially tried to preparatively propagate 0105phi7-2 in 0.12–0.15 % agarose gel-supported medium, a procedure previously successful with other phages [18,23]. Fortunately, we discovered that plaques were easily discernible with *A* = 0.20 and more concentrated agarose gels. The plaques had reasonably sharp edges and decreased in size as *A* increased (Figure 2; *A* is indicated beneath a panel). However, when we attempted to propagate 0105phi7-2 in liquid culture using the medium used for in-gel propagation, no significant increase in titer was observed. That is to say, a gel was needed for propagation, even though plaque size increased as the *A* of a plaque-supporting gel decreased. The reason is not known.

**Figure 3:**
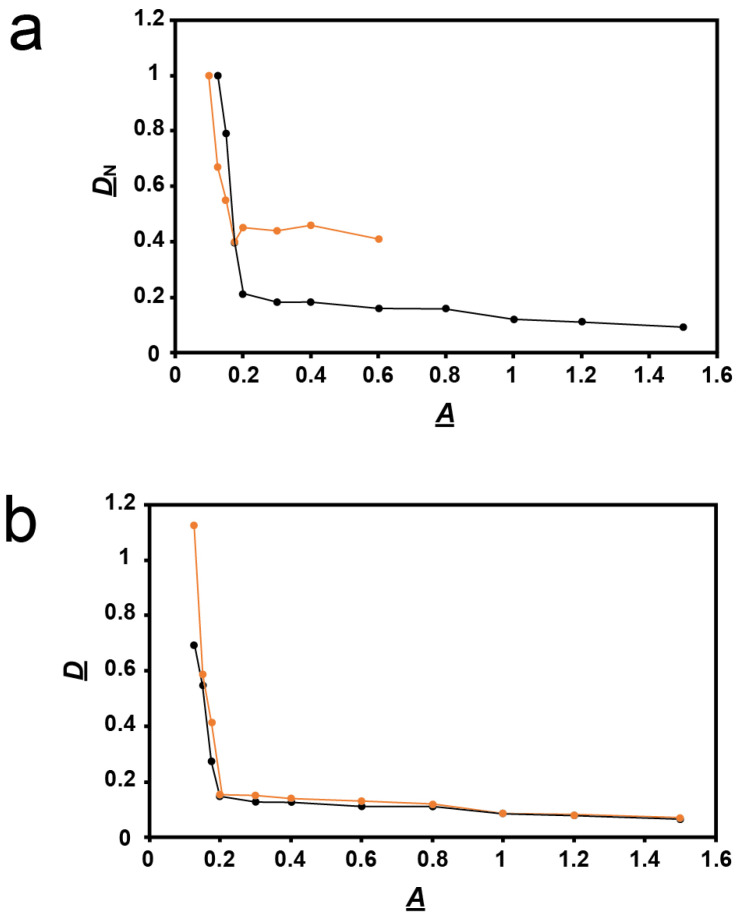
Quantification of plaque size vs. *A*. (**a**) Values of *D*_N_ vs. *A* determined here for phage 0105phi7-2 (orange line), and determined in [18] for phage T4 (black line). (**b**) Values of *D* vs. *A* for phage 0105phi7-2 in the presence of 0.001 M sodium orthovanadate, pH 7.4 (orange line), and in the presence of 0.001 M sodium phosphate, pH 7.4 (black line).

Thus, we performed a more detailed study of plaques formed vs. agarose percentage, *A*. The average diameter of plaques in a supporting agarose gel was called *D*. For comparison with data from a previous study of other phages [18,23], we normalized *D* by dividing it by its value at *A* = 0.1. This ratio is called *D*_N_. For *A* = 0.2 and more, the *D*_N_ vs. *A* plot decreased (black line in Figure 3a) with a slope (average = −0.09 cm^3^/g) that was 117× smaller in magnitude than for *A* between 0.1 and 0.2 (average −10.5 cm^3^/g).

The same plot for phage T4, reproduced from [18], differed significantly. The T4 plot had basically the same shape. However, the T4 plot had the following differences: (1) the slope change at *A* = 0.2 was more gradual, (2) the two slopes, −0.1 and −5.5 cm^3^/g, were less different from each other, and, most diagnostically (the only difference analytically used below), (3) at high *A*, the *D*_N_ values in Figure 3a were 0.4–0.5× lower for 0105phi7-2 than for T4. Although one cannot quantitatively compare absolute plaque sizes, the low-*A* 0105phi7-2 plaques were larger than any T4 plaque ever seen, which explains the relatively low 0105phi7-2 *D*_N_ values. One explanation for difference (3) in *D*_N_ vs. *A* plots was that the sieving of the gel had an effect on T4 that was less than its effect on 0105phi7-2.

However, this interpretation disagrees with the observation that phage 0105phi7-2 is smaller than phage T4, as discussed in Section 3.1. Thus, the 0105phi7-2 slope change at *A* = 0.2 is influenced by a factor that is not present for T4. Nonetheless, slope change at *A* = 0.2 is common to both phages. A likely explanation is presented in Section 3.1.

### 2.3. Characteristics of 0105phi7-2 Plaques at Low A

The plaques for *A* = 0.075–0.15 were large enough that their outlines were sometimes partially or totally lost via plaque overlap, even with ~100 plaques per plate. For *A* = 0.1, *D* was typically >1 cm after 16 h of incubation. For example, in Figure 2, what appear to be remnants of large plaque outlines were present in the 0.1% plaque-supporting agarose gel but were lost via plaque merger in the 0.075% plaque-supporting agarose gel. Further obscuring of plaques was caused by the fact that these plaques were slightly turbid. This plaque-obscuring phenomenon explains the initial difficulty in discerning 0105phi7-2 plaques in ultra-dilute agarose supporting gels.

To determine whether the level of phages present in the slightly turbid regions of 0.10% gels is comparable to the level in a more well-defined plaque, six samples of the upper layer gel were titered. The average was 2.4 × 10^10^ PFU per ml for a 0.10% gel. For samples taken from the clear center of plaques of a 0.40% gel, this average was 8.0 × 10^9^ PFU per ml. Thus, phages were as actively propagating in 0.10% gels, even though plaques were sometimes difficult to discern.

**Figure 4:**
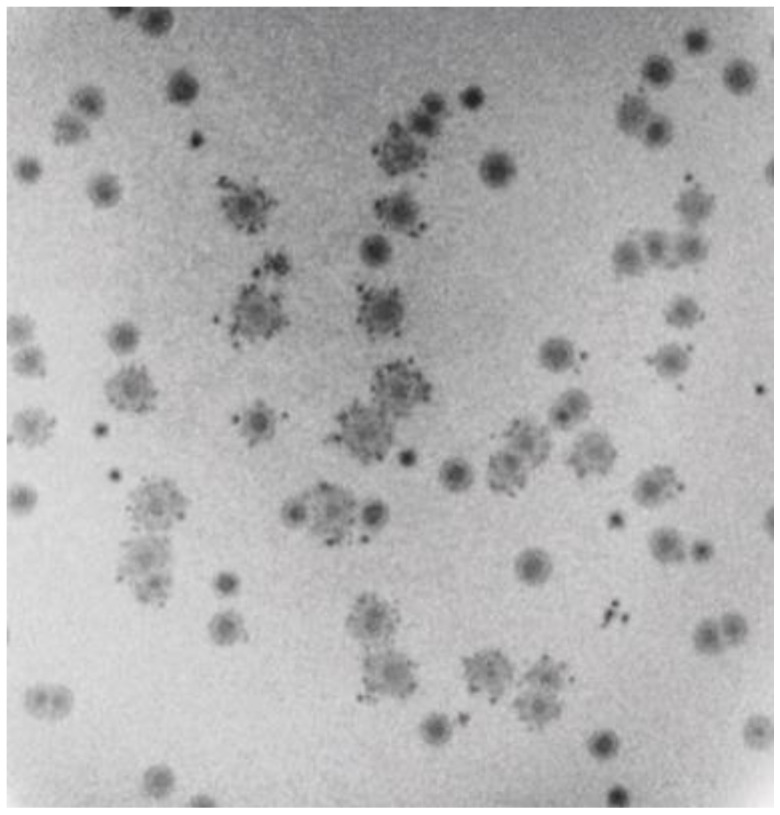
Intermittent satellites of plaques formed in a plaque-supporting gel that has an *A* value of 0.2.

Another unusual feature of 0105phi7-2 propagation was that plaques supported by 0.15–0.20% agarose were each *sometimes* accompanied by multiple, smaller satellite plaques. This was usually seen in 0.15–0.175% plaque-supporting gels. However, a dramatic example, although rarer, was seen in a 0.20% plaque-supporting gel (Figure 4). In Figure 4, satellite-surrounded plaques were found interspersed with plaques without satellites. Plaques of these two types did not arise from two different, comparably abundant phages, as seen by failure to breed true after repropagation from single plaques (not shown). Additionally, no evidence of two different, comparably abundant phages was observed by either electron microscopy (Section 2.5) or genomic sequencing (Section 2.6). Thus, variability of satellite presence was caused by variability in the environment. The satellites were typically 5–6× smaller than the accompanying central plaque. The determinant of the “sometimes” aspect of the satellite plaques is not empirically demonstrated.

In summary, the data of Section 2.1 and Section 2.2 indicate that at least one new phenomenon must be introduced to explain *D*_N_ values and satellite plaques of 0105phi7-2. This phenomenon might be nonthermal motion of extracellular phages, possibly (1) powered by ATP released from host cells, (2) directed to find new hosts and (3) not including attachment to bacteria (i.e., directed phage swimming), which might be inhibited by an ATPase inhibitor.

### 2.4. Testing for Nonthermal Motion: Effects of Orthovanadate during Plaque Formation

**Figure 5:**
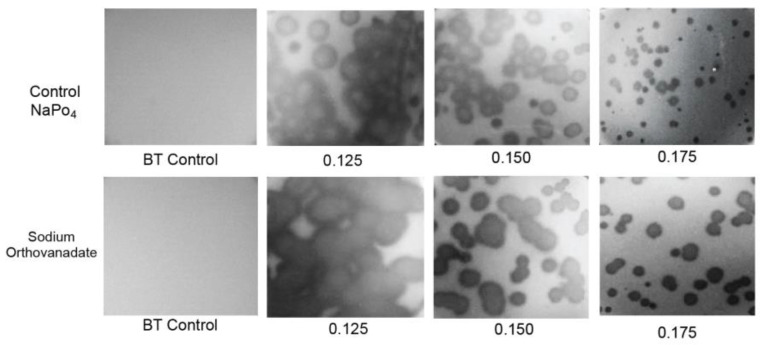
Effects on propagation of sodium orthovanadate. Propagation was performed (top row) after the addition of 0.001 M sodium phosphate to the medium and (bottom row) after addition of 0.001 M sodium vanadate to the medium. All Petri plates were co-incubated and were in thermal contact. *A* values are indicated beneath the panels.

To test for such swimming, *D* vs. *A* was determined in the presence of 0.001 M sodium orthovanadate, a general inhibitor of P-type ATPase activity [24,25] (Figure 5, bottom row; *A* is indicated at the bottom). The plates involved were co-poured and co-incubated in thermal contact with control plates that were used to obtain the *D* vs. *A* plot with phosphate substituted for vanadate (Figure 5, top row). Thus, differences in absolute plaque sizes were significant when one plot was compared to the other at any given *A*. The two plots were, thus, made without normalization of *D*. The addition of orthovanadate had no discernible effect on the turbidity of the lawn (leftmost two panels in Figure 5). Thus, the propagation rate of uninfected bacteria was assumed to be unaltered.

The result was that, in contradiction to the idea of phage swimming, *D* in the presence of orthovanadate (orange line in Figure 3b) was significantly higher than it was for the control (black line in Figure 3b) for *A* = 0.10–0.175. As *A* decreased, this difference became amplified to the point that the orthovanadate+/orthovanadate- *D* ratio approached 2. As *A* increased to 1.2, the difference decreased and was eventually not detected. The results for *A* > 1.0 were obtained in a separate experiment with (1) the same incubation time and (2) scaling based on plaque sizes at overlapping values of *A*.

To test the reproducibility of the orthovanadate-induced plaque size-raising at 0.150 and 0.175%, this comparison was repeated five times each. The orthovanadate-induced plaque size raising was observed in all trials. Orthovanadate increased average plaque size by a factor of 1.14 ± 0.05 for *A* = 0.15 and 1.29 ± 0.17 for *A* = 0.175, i.e., significantly. Furthermore, plaques in the presence of orthovanadate (Figure 5, bottom row) had slightly less internal turbidity than plaques in the controls (Figure 5, top row).

The effect of orthovanadate on plaques is the opposite of what is expected if phages are swimming; the above hypothesis is not correct. A second hypothesis, which includes nonthermal bacterial motion, is given in Section 3.2.

### 2.5. Purification, DNA Sizing, and AGE

**Figure 6:**
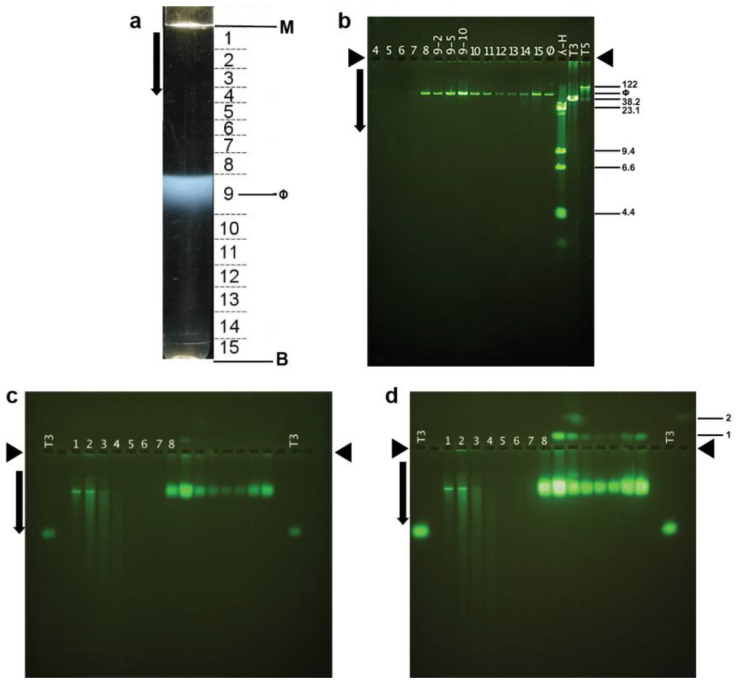
Purification by rate zonal centrifugation, followed by analysis of fractions. (**a**) Light scattering profile of the sucrose gradient (M, meniscus; B, centrifuge tube bottom; horizontal lines, boundaries of fractions as numbered between the lines; ϕ, band formed by phage 0105phi7-2); arrow, direction of sedimentation. (**b**) Agarose gel electrophoresis of DNA expelled from particles in the fractions numbered as in (**a**). (**c**) AGE for 18.0 h, with *A* = 0.6, of particles in the fractions in (**a**), followed by staining with GelStar. (**d**) The gel of (**c**) after expulsion of DNA from phage particles. Length standards in (**b**) are the following (length in Kb indicated at the right): T5 and T3, intact phage T5 and T3 DNAs; λ-H, Hind II digest of phage λ DNA. Lanes in (**c**) and (**d**) have numbers that indicate the fraction in (**a**) that is analyzed. The lane labeled T3 has analysis of purified phage T3. Arrows indicate the direction of electrophoresis; arrowheads indicate origins in (**b**–**d**). The infectivity titers of fractions of the sucrose gradient in (**a**) were the following (fraction number, followed number that produces PFU/mL when multiplied by 10^8^: 1, 0.0091; 2, 0.035; 3, 0.35; 4, 0.30; 5, 0.34; 6, 0.23; 7, 2.51; 8, 1770; 9, 7550; 10, 1080; 11, 443; 12, 210; 13, 215; 14, 528; 15, 1570.

Interpretation of the above results requires knowing the dimensions of phage 0105phi7-2. This information is also basic for understanding and using this phage. To purify 0105phi7-2 phage particles for electron microscopy, initially, phages in a 0.4% agarose gel-plate stock were partially purified by low- and high-speed centrifugation (Section 4.2). The partially purified phages were then more completely purified. We found that we could use either (1) rate zonal ultracentrifugation in a sucrose gradient or (2) hybrid rate and buoyant density ultracentrifugation through a cesium chloride step gradient (Section 3.2). After rate zonal centrifugation in a sucrose gradient, the light scattering profile had a single band (ϕ in Figure 6a). This band was formed by infective phages, based on the PFU/mL of fractions of the sucrose gradient (caption for Figure 6).

The particles in the fractions of Figure 6a all had DNA of unique length, as seen by agarose gel electrophoresis of DNA expelled from capsids (Figure 6b). The length was between 50 and 60 Kb, as judged by comparison with the migration of DNAs of known length in Figure 6b.

**Figure 7:**
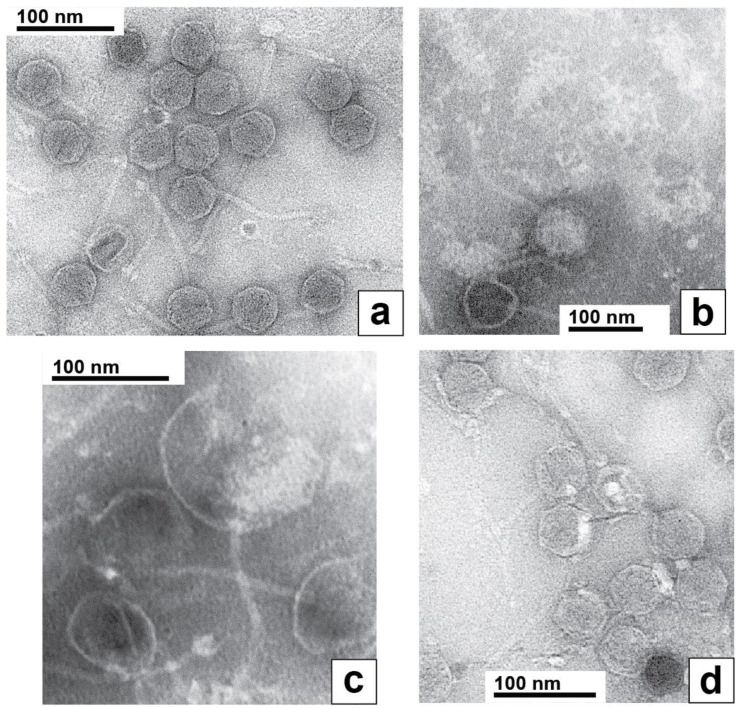
Electron microscopy of negatively stained phage 0105phi7-2. The negative stain was (**a**,**d**) 1.5% uranyl acetate and (**b**,**c**) 1.0% sodium phosphotungstate, pH 8.4.

Electron microscopy of phages revealed that phage 0105phi7-2 was a siphophage, i.e., a dsDNA phage with a relatively long (~150 nm long), noncontractile tail. The heads appeared icosahedral, 50–55 nm in diameter. This was seen (Figure 7a) with the phage negatively stained with 1.5% uranyl acetate. The tip of the tail did not have the straight fiber seen [26,27] on *Escherichia coli* siphophage lambda. Rather, the tail tip had multiple extensions that resembled well-characterized extensions on the tail tip of *Lactobacillus* siphophage TP901-1 [28,29] and *B. subtilis* phage SPP1 [30]. However, no evidence was seen for TP901-1-like [31], whisker-like fibers where the tail and head join. This “whisker” was, in any case, not necessary for infection by phage TP901-1 [31].

Uniquely, to our knowledge among known phages, the entire packaged 0105phi7-2 DNA appeared to retain part of its packaged conformation when spontaneously expelled from its capsid during preparation for EM. This was most dramatically observed during negative staining with 1.0% sodium phosphotungstate, pH 8.4. In this specimen, phages remaining intact had an appearance like the appearance in Figure 7a (filled capsid near, but not at, the bottom in Figure 7b). However, most phages had expelled their DNA genomes, leaving an empty capsid, an example being the empty capsid at the very bottom of Figure 7b. The particles at the top of Figure 7b were the expelled genomes, as judged by their absence when the phage was not in the specimen, their fibrous appearance and their sometimes presence within a broken capsid, as in Figure 7c. These DNA molecules were partially condensed, although the details were probably altered by the negative staining/drying. The likely mechanism of their generation is capsid rupture, rather than DNA injection, because of their sometimes presence in the cavity of ruptured capsids (Figure 7c) and the condensation, which would be lost during injection through a narrow channel. To our knowledge, the above images of DNA are unique in the literature.

To our knowledge, the most similar DNA expulsion is observed with phage G [23,32]. However, unlike the expelled 0105phi7-2 DNA shown in Figure 7b,c, this expelled G DNA had two different conformations, one completely unraveled and the other not distinguishably less compact than it was inside of the phage capsid [23,32].

The partially condensed 0105phi7-2 genomes often appeared roughly conical or rectangular (Figure 7b). The long dimension (axis) was sometimes seen directly to be derived from a packaged DNA axis coincident with the axis of the tail (Figure 7c).

In limited regions of the specimen of Figure 7a, the phages had bright (stain-impermeable), variable shape/size, 4–10 nm objects bound to their heads (Figure 7d). These characteristics of the bright regions are also expected characteristics of host (Gram-positive) cell wall that has been degraded. One interpretation is that the origin of these bright regions is head–host-cell binding observed in Figure 1c.

### 2.6. The Annotated Genome of 0105phi7-2: Proteins of the Mature Phage

**Figure 8:**
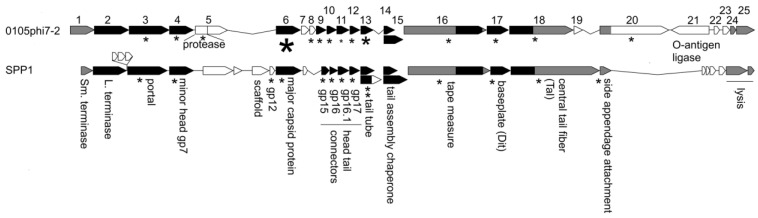
Comparison of the structure and morphogenesis modules of 0105phi7-2 and SPP1. Genes aligned in black are homologous at the HMM-to-HMM comparison level. Genes aligned in gray are inferred to be analogous in function. Proteins known to be structural in SPP1 are marked with an asterisk. Proteins detected by mass spectrometry in 0105phi7-2 are marked with an asterisk scaled to roughly indicate abundance in the virion.

Sequencing, followed by annotation, of the 0105phi7-2 genome revealed a gene cluster with genes that encoded functions needed for assembly of progeny phages. We confidently established homology, through HMM-to-HMM comparisons, to distantly related, well-characterized phages, most closely, *Bacillus subtilis* phage SPP1 (Figure 8). The small (Sm.) and large (L.) terminase genes (gene 1 and gene 2, respectively, for 0105phi7-2) were assigned by HHpred matching to Pfam model PF03592.19, and pdb model 5OE8, respectively, as indicated in the GenBank submission. The protein product of a gene is named gp, followed by the gene number.

This alignment continues until the head assembly region is reached. Although the genes up to and including the gene for a minor protein of the head (gene 4 for 0105phi7-2) align for 0105phi7-2 and SPP1, nonaligned open reading frames are next in the genome, until the gene for the major capsid protein (here, sometimes called major head protein), gp6 for 0105phi7-2, is reached. In the nonaligned region, phage SPP1 encodes a scaffolding protein that assists assembly of the major head protein to form a procapsid that will eventually use terminase proteins to package DNA. Similarly, separate scaffolding proteins are present in some other phages, including phage phi29, P22, SPP1, T4, and T7 (reviews [33,34]). Phages without a separate scaffolding protein have, in the past, been found (1) to have an assembly-promoting peptide fused to the N-terminus of the major capsid (head) protein and (2) to cleavage-remove this peptide, after assembly of a procapsid (HK97 [35]; T5 [36]).

Phage 0105phi7-2 does not have a gene that recognizably encodes a scaffolding protein. The N-terminal region of its gp5 has homology to a prohead protease. However, the N-terminus of gp6 does not have a propeptide predicted to be removed by cleavage.

In the map shown in Figure 8, the genes for assembly of the phage head are followed by genes encoding other capsid-generating functions including assembly of the tail and connecting the tail to the head. These are genes 7–20 for 0105phi7-2. Alignment of this region with the analogous region of SPP1 is good until the region for the head–distal tail tip is reached.

**Figure 9:**
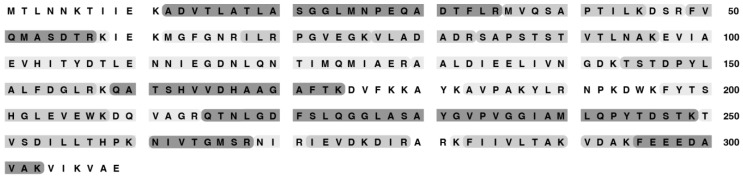
Sequence coverage based on mass spectrometry analysis of gp6 isolated from purified phage 0105phi7-2. The darkness of shading of the indicated peptides corresponds to the relative number of spectra assigned with high confidence.

Mass spectrometry analysis of proteins in purified 0105phi7-2 phage particles was conducted to identify the structural proteins present. The results also permitted characterization of gp6. Detected 0105phi7-2 proteins are indicated by an asterisk in Figure 8. The size of the asterisk corresponds to the number of spectra assigned with high confidence. Gp6 had the largest number of high-confidence spectral counts, with details shown in Figure 9. Assigned spectral counts for gp6 are indicated by shaded sequences in Figure 9, the darker the shading, the larger the number of spectra assigned with high confidence. A large number of gp6 spectra were assigned to the peptide containing amino acids 12–38. K11 was retained on the peptide in a few instances. The lack of sequence coverage of residues 1–10 combined with the large number of spectra assigned to the adjacent peptide indicate that the 10 N-terminal amino acids were not present in the phage particles that were analyzed. In view of the fact that trypsin was used for proteolytic digestion prior to MS analysis, high frequency detection of K11 would not be expected, so its status after maturation cannot be confidently determined. Thus, if head assembly-associated cleavage occurs during assembly (the observed cleavage might have occurred after assembly), it removes a peptide far smaller than those previously observed for phages HK97 (102 amino acids [35]) and T5 (159 amino acids 36]). Thus, 0105phi7-2 is anomalous in its low requirement for scaffolding during head assembly.

The mass spectral analysis of phage 0105phi7-2 also confidently reveals the presence of the (1) major tail protein, gp13; (2) the tail length-determining protein (tape measure protein), gp16; (3) proteins at the tip of the tail, gp17, gp18, and gp20; (4) a protein that forms the portal through which DNA enters the head, gp3; and (5) proteins between the portal and the tail, gp9-12. By use of informatic procedures (caption for Figure 8), gp8 was found to be a homolog of an SPP1 protein at the head–tail interface.

### 2.7. Native Agarose Gel Electrophoresis, and Murine Persistence

**Figure 10:**
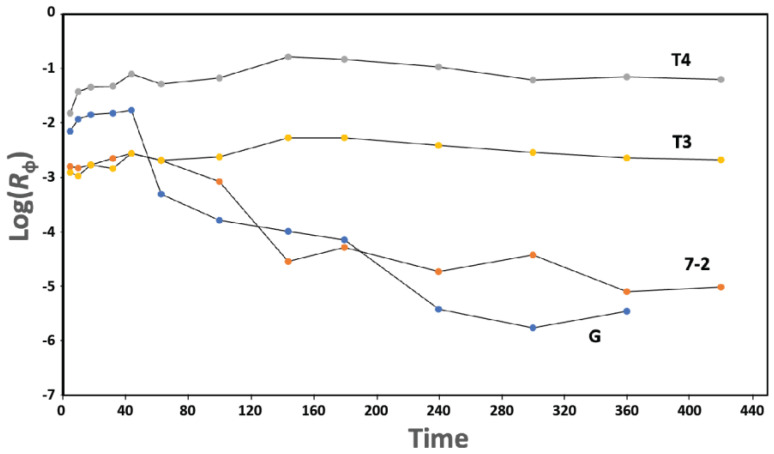
Persistence. After IP inoculation of a mixture of phages T3, T4, 0105phi7-2, and G, the titer and *R*_ϕ_ (Section 4.4) are separately determined for each phage. *R*_ϕ_ values are plotted vs. time (min.) for phages T3 (plot labeled T3), T4 (plot labeled T4), G (plot labeled G), and 0105phi7-2 (plot labeled 7-2).

Long phage lifetime in blood (high persistence) appears to be essential for positive therapeutic outcome when phages are part of therapies, including phage therapy of infectious disease [37]. However, we find phage 0105phi7-2 to be a low-persistence phage (7-2 in the semilogarithmic plot of Figure 10), as is phage G (G in Figure 10), when compared to high-persistence phages. The high-persistence phages are T3 (T3 in Figure 10) and higher persistence phage, T4 (T4 in Figure 10). The various phages were each assayed by separate plating of a dilution of the same blood samples from one mouse.

Biomedical phage use will be assisted by a more efficient (in time and cost) way to rapidly identify and screen out low-persistence phages. We suspect that useful “screening-out” criteria include either a positive σ or a negative σ relatively low in magnitude, as found for the agglutination of red blood cells [38,39,40].

Thus, we determined *E*_T3_(0) (Section 4.3) for phage 0105phi7-2 by use of AGE. Initially, AGE in a 0.6% agarose gel revealed that most particles forming the 0105phi7-2 phage band in the sucrose gradient were negatively charged (anode-migrating). A major AGE-band (sucrose gradient fractions 8–10 in Figure 6c) was formed. The *E*_T3_ was 0.46; phage T3 is in lanes indicated by T3 in Figure 6c. In a separate experiment (not shown), extrapolation produced *E*_T3_(0) of 0.53.

The particles forming the major AGE-band were confirmed to be phages by the following. The major AGE-band increased dramatically in intensity after overnight incubation in 0.002 M EDTA (Figure 6d). This incubation has been found to cause expulsion of packaged DNA [23] and was used as a test for packaged DNA because DNA expulsion has been found [41] to dramatically increase staining relative to unpackaged DNA. Unpackaged DNA formed the sharp band in Figure 6c,d, fractions 1 and 2, a band that underwent only a minor increase in intensity in Figure 6d. Thus, the dominant AGE-band in Figure 6c, fractions 8–10, was formed by phage particles. This conclusion was confirmed by staining of these particles with the protein-specific stain, Coomassie blue (not shown).

Additional, possibly phage-related particles were revealed by the fluorescence intensity increase in Figure 6d. Some of these particles formed a band labeled 1 (top right) in Figure 6d. Band 1 was formed by cathode-migrating particles, indicating a net positive σ. The band 1-particles almost co-sedimented with the negative-σ phages in the sucrose gradient, supporting the designation of both as phages. Slightly more rapid sedimentation for the band 1 particles is discernible via fraction number-dependence of band 1 intensity. That is to say, phage 0105phi7-2 had at least two electrophoretically distinguishable, alternately charged states. This aspect has apparently not been found among other double-stranded DNA phages. In summary, as predicted above, the low persistence of phage 0105phi-7-2 was accompanied by σ that was either positive or low-magnitude negative. More extensive studies of the relationship of persistence to σ are in progress.

The AGE was further enhanced by packaged DNA particles, in minor amount, that formed band 2, as shown in Figure 6d. These particles (Figure 6d, fraction 10) sedimented 1.1× as far as the phage and were even more positively charged than the band 1 particles. Band 2 was also broader than band 1, suggesting particle heterogeneity. The higher sedimentation rate suggested something extra bound to the phage particles, as in Figure 7d. In Figure 7d, the variability of the size and shape of the phage-bound objects suggested that they were pieces of the host envelope. Lesser binding of this type might explain the slightly higher sedimentation rate of the band 1 particles.

## 3. Discussion

### 3.1. D_N_ vs. A Plots: Explanation for the Change in Slope

*D*_N_ vs. *A* plots had a change in slope at *A* = 0.2 for both phage T4, propagated on *Escherichia coli*, and phage 0105phi7-2, propagated on *B. thuringiensis*. Although with a smaller slope change, the same has been found true for a larger phage, 0305phi8-36, also propagated *B. thuringiensis* [18]. This change in slope was not explained in previous studies.

The following observations indicate that this change in slope is primarily an effect of the plaque-embedding gel on the bacterial host, not on the phage. Agarose is chemically inert to proteins and nucleic acids; that is why agarose/agar is used as an electrophoretic and a growth medium. Thus, we assume that the slope change is an effect of sieving. The particle being sieved must be close in size to the size of the effective diameter of pores of the 0.2% Seakem Gold agarose gel (2 × *P*_E_ in [42]), given the abruptness of the slope change. At *A* = 0.2, the value of 2 × *P*_E_ for Seakem LE agarose is 1480 nm [42]. Seakem Gold has a lower electro-osmosis and higher gel strength [43], which indicate an even larger 2 × *P*_E_ at *A* = 0.2 [42]. The high gel strength of Seakem Gold is a major reason for its use in the current study.

Neither phage 0105phi7-2 (head, <65 nm in diameter; tail, 150 nm long) nor phage T4 (head, 90–115 nm in diameter; tail, ~100 nm long [41]) is close in size to the 2 × *P*_E_ of 1480 nm. Thus, the phages are too small to cause the change in slope via sieving.

However, the bacterial hosts are about the right size to cause this change in slope. Both hosts are 500–700 nm wide and 1000–3000 nm long [44]. In fact, when these bacteria propagate in 0.6–0.8% Seakem Gold agarose gels, they are large enough so that, as they grow, they push aside fibers of the gel, as seen by electron microscopy [44]. Thus, we conclude that the increase in slope as *A* decreases below 0.2 (Figure 3) is caused primarily by increase in the freedom of bacterial motion, for both phage 0105phi7-2 and phage T4.

### 3.2. Relationship of the Motion of Bacteria to the Formation of Large Plaques in 0105phi7-2

However, the effect of *A* < 0.20 on plaque size is significantly greater for phage 0105phi7-2 than it is for phage T4, as seen in Figure 3a. The enhancement of phage plaques by ATPase-inhibiting orthovanadate implies that that ATP promotes phage propagation. One possibility is that ATP promotes phage head–host-cell binding, which is suggested by the observed head–host binding described in Section 2.1 (Figure 1c) and supported in Section 2.5 and Section 2.7 (Figure 6d and Figure 7d). The head–host-cell bound phages would be noninfective, until their heads detached from the host cell, for example, when a region of lower ATP concentration is reached.

Thus, we propose the hypothesis that, for *A* below 0.2, (1) host mobility increase is accompanied by ATP-promoted phage head–host cell binding that results in “ride-hitching” on motile host cells, (2) 0105phi7-2 becomes noninfective when head-host bound, and (3) subsequently, at lower ATP concentration, 0105phi7-2 converts to tail–host binding, followed by DNA injection that is not detected in thin sections. This hypothesis explains the turbid strip between satellites and main plaque (Figure 4) as a region through which phages are transported in a mostly noninfective state. Via phage ride-hitching, this hypothesis also explains orthovanadate-induced increase in plaque size.

The orthovanadate also has the potential to counter this stimulatory effect by inhibiting bacterial motility. Thus, the hypothesis includes the assumption that this counter-effect is, sometimes, smaller than the stimulatory effect on ride-hitching. Indeed, the nonuniformity of the presence of satellites (Figure 4) might be explained by inter-plaque variability in the outcome of competition between the stimulatory and counter-stimulatory effect of ATP on ride-hitching.

A subtext is the hypothesis that in-gel propagating bacterial cells undergo nonthermal motion away from relatively high ATP concentrations. This is logical because high ATP concentrations signal the presence of cell-lysing particles. The phage counters by binding to the bacteria. Finally, the positive σ-state of the phage might be the state that is head–host-cell binding, given that the surfaces of bacteria are negatively charged [45,46].

If so, phage 0105phi7-2 must have a history of evolution in a semisolid medium that does not flow, i.e., a biofilm [47,48]. Otherwise, fluid flows, not cellular activities, are the dominant determinants of the positions of phages and host cells.

### 3.3. Bacterial Host and Conditions of Propagation

The above hypothesis is consistent with the observation (Section 2.3) that phage 0105phi7-2 does not propagate in liquid culture, in that, by this hypothesis, phage 0105phi7-2 requires a biofilm-like (i.e., a gelled [47,48]) environment. This environment is provided here by an agarose gel. Our original use of in-gel propagation was designed, in part, to increase the probability of isolating biofilm-inhabiting phages.

*B. subtilis* phage SPP1 [49,50] and *Lactobacillus* phage TP901-1 [51] were chosen as prototypes for comparison of the structural proteins of 0105phi7-2 because they have been characterized by cryo-EM and thus provided detailed information about the role of each protein in the phage structure.

These are, however, not close relatives of 0105phi7-2, based on genomic sequence. They represent a very large section of siphophages, hosted by Gram-positive bacteria, with similar structure. BlastP searches of NCBI nr with 0105phi7-2 protein sequences reveal (1) at least nine isolated phages of *B. thuringiensis* that are much closer to 0105phi7-2 in a mosaic pattern of similarity and (2) innumerable matches to proteins from segments of *B. thuringiensis* chromosomes, segments that are presumptive prophages or prophage remnants. The extent to which the unusual growth properties of 0105phi7-2 are distributed in the population of close relatives is unknown.

### 3.4. Physical and Chemical Phage Properties: Assembly

Phage 0105phi7-2 is found here to have two properties that can help simplify analysis of phage DNA packaging. First, the partially decondensed genomes provide improved access to the conformation of the packaged genome. For example, the axis observed in images of these genomes (Figure 7b,c) implies a similar axis of the fully packaged genome and also that this axis (1) is coincident with the channel of DNA entry and (2) formed without assistance of additional coaxial internal proteins, such as those found [52] in phages T3 and T7.

The second property is low assembly assistance for gp6 (no scaffolding protein; minimal, if any, N-terminal peptide removed during assembly). The only apparent example of comparably short peptide removal is the removal of an approximately 14-amino acid peptide from the N-terminus of the major head protein of siphophage 80a of *Staphylococcus aureus* [53]. However, a scaffolding protein was present in this case. This property of 0105phi7-2 suggests a lower conformation-based energy barrier between the procapsid and mature capsid states of the major head protein. Reasoning by analogy with coliphage P2 [54], 0105phi7-2 scaffolding might be generated by a peptide released from the C-terminal region of gp5 by the N-terminal protease activity of gp5.

Relatively low energy-barrier transitions of this protein can also explain the finding of two electrophoretically (σ)-defined states of the mature phage. A similar finding, though with less difference in σ-values, has been made for phage T7. In the case of T7, both states had negative σ and phages in the least negative-σ state were more adsorptive to host cells [55]. Flexibility for T7 state interconversion appears to be assisted by the presence of major head protein molecules of two different lengths [55].

Both electrophoretic states of 0105phi7-2 are relatively low in negative surface charge, which is reasonably thought to promote low blood persistence. Low murine blood persistence is, indeed, found here for 0105phi7-2 (Figure 10). More work is needed to determine how completely low persistence is correlated with a relatively low component of negative surface charges. Efficient screening out of low-persistence phages, possibly based on σ, is expected to improve phage therapy of infectious disease [37]. Thus, this work appears to be at the top of the priority list for phage therapy.

### 3.5. Genome: Orfans

Analysis of the complete genome of 0105phi7-2 in the GenBank submission also reveals that about 29% of the 52.629 Kb genome is in a single block (orf47-orf87) enriched for orfs that encode proteins of unknown function (orfans). This includes most of the rightwards transcript before the structure/morphogenesis module.

One way to organize thinking about the function of orfans is to assume that function is correlated with position in the genome. Analysis of orfan-annotated gene neighbors in several different phage sequences, without any biochemical analysis of orfan gene products, has led to the conclusion that small gene orfans of phage T4 have a function linked to that of neighboring genes for proteins of the T4 base plate [56]. This logic implies that 0105phi7-2 genes 7 and 8 have functions in assembly, although these genes are not otherwise annotated.

Finally, the complete map of the 0105phi7-2 genome has the following genes that suggest that 0105phi7-2 has history as a temperate phage: immunity repressor homologs, orfs29, 40; anti-repressor protein homolog, orf 46. We do not have direct evidence that 0105phi7-2 is currently temperate.

### 3.6. Implications for Future Isolation of Phages

In relation to the behavior of phages isolated in the past (including the phages that we have isolated), the isolation of phage 0105phi7-2 is eccentric. The following question arises. Can we obtain a complete understanding of phage evolution and phage biomedical potential without managing phage propagation eccentricities? When one considers that (1) metagenomic analysis has identified phages with 634, 636, 642, and 735 kb genomes [57] and that, among isolated/propagated phages, only phage G has a comparably sized genome [58], the likely answer is no.

Additionally, phage G was isolated in Italy 54 years ago [58] as part of what is logically described as a freak accident [59,60]. We have attempted (and presumably others have also) to isolate phages with either longer or comparably long genomes, without success, even though we have demonstrated that dilute agarose gels accommodate them [23]. Also significant in this area is the fact that phage G was almost lost in ~year 2000. Phage G was saved from extinction by the efforts of S.J. Hayes who retrieved what is today wild-type phage G from an almost completely dead preparation. A second trying episode occurred about five years ago when we confirmed that phage G did not survive prolonged freezing. The bottom line was that phage eccentricities were not to be avoided, but embraced, if we were to progress.

A key aspect for the future is the speed of phage isolation and characterization. Most phages isolated are expected to be either too “not novel” or too “not useful” to further pursue in detail. High speed of isolation and characterization is essential to move through the forest of existing phages. In-gel propagation helps for this purpose, the rate of isolation being capable of exceeding 50 phages per week per person. Additionally, determining *D* vs. *A* helps to start the characterization. Electron microscopy of plaques can further help, as shown above. A key objective for the future is increasing the efficiency, in time and cost, of the characterization of phages for various purposes, including phage therapy of infectious disease.

## 4. Materials and Methods

### 4.1. Propagation of Phages

Phages 0105phi7-2 and 0305phi7-4 were propagated on the host, *Bacillus thuringiensis* [18], in a gelled agarose overlay, by use of procedures previously described [17,18]. The overlay had the following medium: 10 g tryptone, 5 g KCl in 1 L of water (T broth). Overlay gelation was achieved by including Seakem Gold agarose (Lonza, Rockland, ME, USA). The concentration of agarose is stated in the text below. Underneath the overlay was gel formed by 1.0% agar (Fischer Scientific, Waltham, MA, USA) in the same medium. Phage T3 was propagated in liquid culture by use of procedures previously described [61]. Phages G and T4 were propagated in-gel and purified by procedures previously described (T4 [41]; G [23]).

### 4.2. Purification of Phages and Capsids

Phages and phage capsids were partially purified by use of high- and low-speed centrifugation. First, preparative propagation was performed, in an agarose overlay (plate stock) prepared as described in Section 4.1, with 0.4% agarose, and a phage inoculum of 3.2 × 10^5^ plaque-forming units (PFU). The number of 15 cm Petri plates was six. These plates were incubated at room temperature 25 ± 3 °C for 16 h. Phages were then extracted by (1) removing the upper layer gel with a spatula, (2) adding medium without agarose (2.0 mL per 15 cm Petri plate) and then vortexing.

Next, cells, cell fragments, and agarose gel were removed by pelleting via (low-speed) centrifugation at 7000 rpm for 10 min, at 5 °C, in a JLA-16.250 rotor in a Beckman Avanti J-25 centrifuge (maximum g = 7879). The supernatant was collected, and the resulting pellet was re-extracted twice by resuspending in agarose-free medium, followed by re-pelleting at low speed. After pooling of supernatants, phages and related particles were pelleted by centrifugation at 20,000 rpm for 6.0 h, at 5 °C, in a JA-25.50 rotor in a Beckman Avanti J-25 centrifuge (maximum g = 51,840). These pelleted, partially purified particles were resuspended in 0.2 P-M buffer: 0.2 M NaCl, 0.01 M sodium phosphate, pH 7.4, 0.001 M MgCl_2_.

Partially purified particles were further fractionated by use of either one or both of the following procedures, as indicated below: (1) rate zonal centrifugation in a sucrose gradient and (2) density gradient centrifugation in a cesium chloride step gradient, a procedure that fractionates by both rate zonal and buoyant density centrifugation. The following was the procedure for (1). A 10–35% sucrose gradient was pre-poured, above a 0.7 mL, 62% sucrose layer, in a centrifuge tube for the Beckman SW41 rotor. All sucrose solutions were in the following buffer: 0.01 M Tris-Cl, pH 7.4, 0.01 M MgSO_4_, 6% polyethylene glycol 3350. A sample with a volume of 0.5 mL was layered on top of the sucrose gradient. The sample was centrifuged at 25,000 rpm, at 5 °C, for 2.25 h in a Beckman SW41 rotor (average g = 114,825). A digital photograph was taken of light scattered from particles in the sucrose gradient. Fractions were collected by pipetting from the top with visual detection of bands of light scattering.

The following was the procedure for (2), above. A CsCl gradient was pre-poured in the following steps in a centrifuge tube for the Beckman SW55Ti rotor (volume, followed by refractive index-determined density [g/mL]): 1.0 mL, 1.1438; 0.8 mL, 1.2052; 0.6, 1.2985; 0.9, 1.4247; 0.9, 1.5964. The buffer was 0.01 M Tris-Cl, pH 7.4, 0.01 M MgSO_4_. A 0.4 mL portion of sample was mixed with 0.6 mL of 0.01 M Tris-Cl, pH 7.4, 0.001 M MgCl_2_. This mixture was layered on the CsCl step gradient and centrifuged at 5 °C, 38,000 rpm, for 2 h. Fractions were collected by pipetting from the top with visual detection of bands. The band formed by phages was identified by infectivity titer and electron microscopy, the latter as described below.

### 4.3. Native Agarose Gel Electrophoresis (AGE): Relative Values of Average Electrical Surface Charge Density (σ)

Native (intact phage particle) agarose gel electrophoresis (AGE) was performed in submerged, horizontal agarose slab gels, by use of procedures previously described [62], with the agarose percentage, *A*, indicated below (Seakem Gold agarose). The electrophoresis buffer was 0.09 M Tris-Acetate, pH 8.4, 0.001 M MgCl_2_. The buffer was circulated to prevent the formation of a pH gradient. Temperature was 25 °C and was controlled ± 0.3 °C by circulation through a temperature-controlled water bath.

Gels were stained post-electrophoresis with GelStar, a fluorescent dye (Lonza). The dye, as purchased, was diluted by 1/200 into electrophoresis buffer in which the gel was submerged. Staining continued for 2 h after which a digital photograph was taken. To expel packaged DNA and, therefore, increase staining to an extent greater than for unpackaged DNA, a gel was incubated at room temperature overnight in 0.002 M EDTA, pH 7.4 [41]. Finally, some gels were subsequently protein-stained with Coomassie blue [63].

To standardize the distance migrated by 0105phi7-2, this distance was divided by the distance migrated in the same gel by phage T3 (*E*_T3_). To remove effects of particle size and leave only effects of average electrical charge density (σ), *E*_T3_ was extrapolated to an *A* of 0 [62], which is called *E*_T3_(0). A value of *E*_T3_(0) can be converted to electrophoretic mobility, extrapolated to *A* of 0, by multiplying by the extrapolated electrophoretic mobility of phage T3, which is 1.5 × 10^−4^ cm^2^/(V.min), in the electrophoresis buffer used here.

### 4.4. Determination of Persistence

Phage persistence in murine blood was determined with the following procedure. An intraperitoneal (IP) phage injection of 160 μL was performed with a mixture of phages 0105phi7-2, G, T3, and T4 into a 11–12 week-old female C57/BL6 mouse. The initial titers (PFU/mL) were 0105phi7-2, 1.0 × 10^11^; G, 9.9 × 10^9^; T3, 2.8 × 10^11^; T4, 3.5 × 10^10^. The mixture had been made in 0.2 M NaCl, 0.01 M Tris-Cl, pH 7.4, 0.001 M MgCl_2_. After IP injection, 1 μL samples of blood were taken from the tail vein at the times indicated. The phages in these samples were immediately diluted into 1 mL of T broth. PFU per ml were assayed, with plating that started within 1.0 h of blood sampling. Conditions of plating phages G and 0105phi7-2 were those used for preparative propagation in Section 4.1. The conditions for plating phages T3 and T4 are described in [37].

For each phage, the following ratio (*R*_ϕ_) was determined: titer (PFU/mL) at each blood sampling time, divided by the total number of PFU inoculated. *R*_ϕ_ was plotted vs. time. T3 and T4 were already known [37] to be relatively high-persistence phages. T3 plaques were distinguished from T4 plaques by use of hosts and plating conditions that plated either T3 or T4 separately. Details are previously described [37]. Phage G and phage 0105phi7-2 both plate on hosts that do not plate the other phages in the mixture.

### 4.5. Agarose Gel Electrophoresis of DNA

To perform agarose gel electrophoresis of DNA expelled from particles in fractions of a sucrose density gradient, the following was performed. To digest unpackaged DNA, 1 μL of 750 mg/mL of DNase I was added to 15 μL of the fraction and incubated for 1.0 h at 30 °C. The digestion was stopped by adding the following: (1) 9 μL of 0.1 M NaCl, 0.01 M Tris-Cl, pH 7.4, 0.002 M EDTA and (2) 5 μL of 30% Sucrose, 0.6 M NaCl, 0.06 M Tris-Cl, pH 7.4, 0.06 M EDTA, pH 7.4, 6% Sarkosyl, 1.2 mg/mL bromophenol blue. DNA was expelled by incubation in an 85 °C water bath for 10 min. The DNA samples were kept on ice until loaded.

To fractionate the expelled DNA, together with linear DNA standards of known length, electrophoresis was performed in a 0.4% Seakem Gold agarose gel. The gel was cast in and submerged beneath the following buffer: 0.09 M Tris-acetate, pH 8.4, 0.01 M EDTA. Then, sample mixtures were layered in sample wells with a 50 μL glass micropipette. Electrophoresis was performed at 0.55 V/cm, 25 ± 3 °C for 16.0 h. Post-electrophoresis staining was performed with GelStar, using the procedure described in Section 4.3.

DNA standards used included the following: a Hind III digest of mature phage λ DNA (48.5, 27.5, 9.4, 6.6, 4.4 Kb [64]), phage T3 DNA (38.2 Kb [65]), phage T5 DNA (122 Kb [66]), the latter two expelled from phages (preparation of phage T5 [42]), as described above.

### 4.6. Electron Microscopy

Electron microscopy of thin sections was performed by use of procedures previously described [21]. The specimens were 0.4% agarose gel-supported plaques, sampled with a 100 μL glass micropipette, so that the center of a specimen was the border between the clear region of the plaque and the turbid surrounding region.

Samples of purified phage 0105phi7-2 were negatively stained for electron microscopy by use of the following procedure. A sample of a gradient fraction was (1) absorbed to a carbon support film for 2 min, (2) washed with 5 drops of MilliQ-purified H_2_O (MilliQ/Sigma, Burlington, MA, USA), and then (3) washed with 3 drops of either 1.5% uranyl acetate or 1.0% sodium phosphotungstate, pH 8.4. The excess stain was removed by wicking with Whatman filter paper #1. The sample was air-dried for at least 15 min.

All specimens were observed in a JEM-1400 electron microscope, operated at 80 kV, in the Department of Pathology, UT Health, San Antonio. Images were recorded with an AMT image capture engine (Version 7).

### 4.7. Sequencing and Annotation of the 0105phi7-2 Genome

For sequencing of the 0105phi7-2 genome, 0105phi7-2 DNA was phenol-extracted from phages that had been (1) purified by centrifugation in a cesium chloride step gradient and (2) then dialyzed against 0.1 M NaCl, 0.001 M Tris-Cl, pH 7.4, 0.001 M EDTA. Genomic DNA was quantified, and a genomic DNA-seq library preparation was made, with an Illumina Nextera XT library preparation kit by following Illumina protocol (Illumina, San Diego, CA, USA). The DNA-seq library was sequenced using Illumina NextSeq 500 System with the sequencing module 75 bp single read sequencing. Approximately 5.5 million reads were generated after demultiplexing. The sequence was obtained at the Greehey Children’s Cancer Research Institute of UT Health, San Antonio.

The genome was annotated with the following procedure. Predictions of reading frames were obtained by GeneMark [67] from the web server at http://opal.biology.gatech.edu/GeneMark/ (accessed on 17 May 2023). All predicted frames were searched by Psi-Blast [68] against the NCBI nr database. If multiple start codons were predicted by GeneMark, the start codon was selected to include all detected similarity with the multitudinous Psi-Blast matches to phages and prospective prophages in nr. Frames were functionally annotated through use of the HHpred server [69] at https://toolkit.tuebingen.mpg.de/tools/hhpred (accessed on 17 May 2023), with special attention to matches including the prototypical siphoviruses SPP1 and TP901-1, as outlined by [26]. HHpred models matched are indicated in the GenBank submission OQ317942. Graphics were created by the system described in [70].

### 4.8. Mass Spectrometry Analysis of Capsid Proteins

We used mass spectrometry to determine which phage genome-encoded proteins were in phage particles obtained after purification by rate zonal centrifugation in a sucrose gradient. The procedure was the following. A sample aliquot from sucrose gradient centrifugation was mixed with 5% SDS in 50 mM triethylammonium bicarbonate (TEAB). Proteins were reduced with tris(2-carboxyethyl)phosphine hydrochloride (TCEP), alkylated in the dark with iodoacetamide, and applied to an S-Trap (Protifi) for tryptic digestion (sequencing grade; Promega) overnight in 50 mM TEAB. Peptides were eluted from the S-Trap with 0.2% formic acid in 50% aqueous acetonitrile and quantified using Pierce™ Quantitative Fluorometric Peptide Assay (Thermo Scientific). The digest was analyzed by capillary HPLC-electrospray ionization tandem mass spectrometry on a Thermo Scientific Orbitrap Fusion Lumos mass spectrometer. On-line HPLC separation was accomplished with an RSLC NANO HPLC system (Thermo Scientific/Dionex, Waltham, MA, USA): column, PicoFrit™ (75 μm i.d.; New Objective, Littleton, MA, USA) packed to 15 cm with C18 adsorbent (Vydac; 218 MS 5 μm, 300 Å); mobile phase A, 0.5% acetic acid (HAc)/0.005% trifluoroacetic acid (TFA) in water; mobile phase B, 90% acetonitrile/0.5% HAc/0.005% TFA/9.5% water; gradient 3 to 42% B in 30 min; flow rate, 0.4 μL/min. Precursor ions were acquired in the orbitrap in centroid mode at 120,000 resolution (*m*/*z* 200); data-dependent higher-energy collisional dissociation (HCD) spectra were acquired at the same time in the linear trap using the “top-speed” option (30% normalized collision energy). Other MS scan parameters included: mass window for precursor ion selection, 0.7; charge states, 2–5; dynamic exclusion, 15 sec (±10 ppm); intensity to trigger MS2, 50,000. Mascot (v2.8.1; Matrix Science) was used to search the spectra against a combination of the following protein sequence databases: Phage_0105phi7-2_20220810 (88 sequences; 15,637 residues); UniProt_Bacillus_thuringiensis_ATCC13367_20230126 (5920 sequences; 1,689,040 residues); common contaminants (124 sequences; 62,564 residues). Cysteine carbamidomethylation was set as a fixed modification and methionine oxidation and deamidation of glutamine and asparagine were considered as variable modifications; trypsin was specified as the proteolytic enzyme, with one missed cleavage allowed. Post-processing of the Mascot results and determination of protein and peptide identity probabilities were accomplished by Scaffold (v5.2.2; Proteome Software) and Scaffold Quant (v5.0.3; Proteome Software). The thresholds for acceptance of peptide and protein assignments in Scaffold Quant were set to achieve a protein level FDR of <1% with a minimum of two peptides required.

## Data Availability

The data presented in this study are all available within one or more of the following: Figure and Text.
